# The clinical characteristics and genotype analysis of *LAMB2* gene mutation

**DOI:** 10.3389/fmed.2024.1437881

**Published:** 2024-10-02

**Authors:** Guangbo Li, Dequan Su, Cuihua Liu, Guanghai Cao, Zhuqin Zhan, Jianying Liao

**Affiliations:** ^1^Department of Nephrology, Fudan University Affiliated Children's Hospital Xiamen Hospital (Xiamen Children's Hospital), Xiamen, China; ^2^Nephrology Department, Henan Provincial Children's Hospital, Henan, China

**Keywords:** LAMB2 gene mutation, steroid-resistant nephrotic syndrome, clinical V manifestations, pathology, genetic mutation features

## Abstract

**Purpose:**

To report a case of steroid-resistant nephrotic syndrome caused by a *LAMB2* gene mutation, examine the associated literature, outline the clinical and genetic features of Pierson syndrome, and deepen the clinical comprehension of this condition.

**Method:**

The study involved retrospective summary and analysis of the clinical presentations, genetic mutation features, and prognosis of one case involving a *LAMB2* gene mutation. PubMed, Medline, Web of Science, CNKI, and Wanfang databases were searched to gather and summarize information on the pathological phenotypes and genotypic alterations associated with *LAMB2* mutations.

**Result:**

A 9-month-old infant presented with edema and massive proteinuria, along with horizontal nystagmus and miosis, manifesting clinically as steroid-resistant nephrotic syndrome. Ocular symptoms prompted both a kidney biopsy and genetic testing. The biopsy revealed minimal change disease, while genetic testing identified compound heterozygous mutations in the *LAMB2* gene: c.1405C > T (p.R469X) and c.1066 T > A (p.C356S), inherited from the father and mother, respectively. These mutations were determined to be novel. The diagnosis was confirmed as a *LAMB2* gene mutation. A literature review of 26 cases with *LAMB2* mutations indicated these typically presented as steroid-resistant or congenital nephrotic syndrome, with 14 cases also displaying ocular symptoms. Among the 18 cases undergoing kidney biopsy, findings included focal segmental glomerulosclerosis in 10 cases, minimal change disease in 4 cases, diffuse mesangial sclerosis in 2 cases, IgM nephropathy in 1 case, and mesangial proliferation in 1 case. Electron microscopy in 10 cases showed basement membrane splitting. Genetic analysis revealed 15 cases with compound heterozygous mutations, 5 with homozygous mutations, 3 with heterozygous mutations, 2 with frame-shift mutations, and 1 with a truncating mutation. 16 out of the 26 reported cases progressed to end-stage kidney disease.

**Conclusion:**

Mutations in the *LAMB2* gene primarily manifest as steroid-resistant or congenital nephrotic syndrome, often accompanied by ocular abnormalities, suggesting a strong likelihood of this disease. The results of genetic testing offer a foundational basis for clinical diagnosis. The identification of a new mutation site in this case expands the known spectrum of mutations in the *LAMB2* gene. Unfortunately, the prognosis associated with this condition is generally poor.

## Introduction

The *LAMB2* gene mutation was initially identified by Pierson in 1963 as a condition affecting both the kidneys and eyes, characterized by congenital nephrotic syndrome and non-reactive small pupils (1). *LAMB2* was localized to chromosome 3p21, and encodes the basement membrane protein laminin beta2, which is incorporated in specific heterotrimeric laminin isoforms and has an expression pattern corresponding to the pattern of organ manifestations in Pierson syndrome. The most deleterious missense mutations that have been identified affect primarily the N-terminus of laminin beta2, biochemical functions of laminin beta2 variants influencing glomerular filtration that may underlie the pathogenesis of isolated nephropathy caused by *LAMB2* abnormalities (1–3). Steroid-resistant nephrotic syndrome (SRNS) is a heterogeneous disease that includes both immune-based genes and a monogenic etiology (4). More than 85% of patients with nephrotic syndrome respond to corticosteroids, but about 10–15% still do not respond to steroids or develop steroid resistance (5)^.^ The median age of onset of SRNS is 4.4 years, with an early age of onset concentrated in early childhood (5). Known as Pierson syndrome, this autosomal recessive genetic disorder accounts for approximately 2.5% of nephrotic syndrome cases in infants under 1 year old, typically presenting within the first 3 months of life (6). The common symptoms include nephrotic syndrome along with neurodevelopmental issues such as hypotonia, muscle weakness/myasthenia, and typically small pupils in terms of ophthalmological signs. The most severely affected infants often do not survive beyond the first year of life, whereas those with less severe forms may progress to chronic kidney failure by age 10 years old (6). Pierson syndrome may also manifest solely as nephrotic syndrome without neurodevelopmental or eye abnormalities, often due to missense mutations, with onset during childhood. Currently, there is no specific treatment for Pierson syndrome, and care primarily involves supportive management.

## Research subject and methodology

### Research subject

This retrospective study focuses on a 1-year-old child with nephrotic syndrome due to a *LAMB2* gene mutation, who was admitted to our hospital in January 2023. The research includes collecting data on the patient’s gender, age, medical history, clinical symptoms, and laboratory results for analysis. The study received approval from the Ethics Committee of Xiamen Children’s Hospital, and informed consent was obtained from the patient’s guardian through the signing of a consent form.

### Whole exome sequencing

The genomic DNA was extracted from peripheral blood, randomly fragmented and sheared into fragments of 180–280 bp in length using a Covaris crusher. Genomic DNA fragments were enriched using the Agilent SureSelect XT Human All Exon V5 kit. After enrichment, DNA libraries were sequenced with the HiSeq 2000 platform according to the manufacturer’s instructions (Illumina, San Diego, CA) with an average on-target sequencing depth of 120×. More information about sequencing and data analysis, particularly of single nucleotide variations, can be found in a previous study. The libraries were linearly amplified by PCR and subjected to library quality tests. After passing the test, they were carried out high-throughput deep sequencing on the Illumina HighSeq 2,500. Burrows-Wheeler Aligner software (version 0.59) was used to align the sequencing reads with the GRCh37.p10. Then the aligned reads were realigned and recalibrated by GATK Indel Realigner and the GATK Base Recalibrator, respectively.^1^ GATK UnifiedGenotyper (see text footnote 1) was used for identifying the single-nucleotide variants (SNV) and small insertions or deletions (indel). Finally, Consensus Coding Sequences Database (20130630) was used for variant annotation. According to the minor allele frequency with a cut-off value of <0.05 in four databases (dbSNP, HapMap, 1,000 Genome Project and in-house Chinese local database), we filtered and selected the variants. Classification of variants (pathogenic, likely pathogenic, variant of uncertain significance, and likely benign and benign) has been done according to the variant interpretation guidelines of American College of Medical Genetics and Genomics (ACMG). Finally, we compared the variants found in patient and other affected and unaffected (carrier or non-carrier) family members. Gene function has been established from the previously published articles and OMIM database (7–9).

### Sanger sequencing

Sanger sequencing was performed to validate the variants identified by whole exome sequencing. Sanger sequencing was performed with these primers: The *LAMB2* variants that were identified with next-generation sequencing [c.1405C > T (p.R469X) and c.1066 T > A (p.C356S)] were confirmed in the proband and her parents by PCR and Sanger sequencing as previously described. The following variant-specific primers were designed through Primer3 online: Intron 20 primers, F: 5′-TGAAAGGTGAGACTGGAGCA-3′ and R: 5′-GAACCCCAATTCAGCCATGC-3′; and Exon 24 primers, F:5′-GTTGCAGTGCCATGGTGAG-3′ and R: 5′-CCAATTTCACGC CTGCAATG-3′.

### Literature retrieval

The relevant literature was retrieved from the PubMed, Medline, Web of Science, CNKI, and Wanfang databases for the years 2004–2024, using the search terms “*LAMB2* gene mutation” and “Pierson syndrome” A total of 148 articles were initially screened. After removing duplicates using Endnote software, 88 articles were selected for further review. Upon full-text reading of the relevant literature, 20 articles specifically related to children with *LAMB2* gene mutations were identified, analyzed, and summarized in Table 1.

**Table 1:** Mutant phenotype and genotype alteration of the *LAMB 2* gene as reported in the literature.

Case	Phenotype			Genotype					
	Age of onset	Age of ESRD	Extrarenal manifestations	Genome (hg19)	Exon	Genetic mutations	Protein	Variation classification	Mutation type
1 (15)	6 M	–	–	Chr3:49169948 Chr3:49163854	2 16	c.225delC c.2095G>C	p.Y76Tfs*36 p.G699R	Pathopoiesia Clinical significance unknown	Compound heterozygous
2 (15)	1 M	–	–	Chr3:49169948 Chr3:49149948	2 16	c.225delC c.2095G>C	p.Y76Tfs*36 p.G699R	Pathopoiesia Clinical significance unknown	Compound heterozygous
3 (16)	3Y	16Y	–	Chr3:49169936 Chr3:49159710	2 28	c.235-237delGTC c.4667C>T	P.A1156V	Pathopoiesia Clinical significance unknown	Compound heterozygous
4 (17)	8 M	–	Nystagmus Bilateral small corneal atrophy	Chr3:49163403 Chr3:490443	11 27	c.1503-1504delAT c.4267delT	p.C502X p.C1423VfsX28	Pathopoiesia Pathopoiesia	Frame-shift
5 (18)	5Y	–	Visual impairment, Optic nerve dysplasia	Chr3:49168561 Chr3:49160880	7 25	c.737G > A c.3982G > C	p.R246Q p.G1328R	Clinical significance unknown Clinical significance unknown	Compound heterozygous
6 (19)	52D	90D	Epidermolysis bullosa	Chr3:49166094	14	c.1890G > T	p.Q630H	Clinical significance unknown	Homozygous
7 (16)	1 M	3 M	Visual impairment	Chr3:49159392 Chr3:49159761	29 28	c.4907-4908delAG c.4616G > A	p.E1636afsX22 p.R1539Q	Pathopoiesia Clinical significance unknown	Truncated mutation
8 (20)	3 M	1Y	Nystagmus	Chr3:49168248 Chr3:49160649	26 8	c.4140C > A C.961 T > A	p.N1380K p.F392del	Clinical significance unknown Clinical significance unknown	Compound heterozygous
9 (21)	34D	4 M	–	Chr3:49167711 Chr3:49159375	9 29	c.4140C > A c.1176-1178delTCT	p.N1380k p.F392del	Clinical significance unknown Clinical significance unknown	Compound heterozygous
10 (22)	6D	1 M	Small pupil	Chr3:49166536 Chr3:49160191	14 27	c.1648C > T c.4519 > T	p.R550X p.E1507x	Pathopoiesia	Compound heterozygous
11 (23)	2 M	3 M	Small pupil Right, underdeveloped eyeball	Chr3:49150139 Chr3:49167663	30 9	c.5077-5088insCCAG c.1225 + 1G > A	p.G1639afs*8	Pathopoiesia	Compound heterozygous
12 (24)	39D	74D	Small pupil Reduced muscle tone	Chr3:49163904–49,163,905	16	c.2044-2045ins TT	p.G682Ffs*13	Pathopoiesia	Homozygous
13 (25)	7D	28D	Small pupil Reduced muscle tone	Ch3:49162353	21	C.2890C > T	p.R964X	Pathopoiesia	Homozygous
14 (26)	1D	63D	Small pupil	Chr3:49160887–49,160,888 Chr3:49160191	25 27	C.2283-2286delCTCT C.3974-3975insA	P.s762RfsX28 P.N1325KfsX1331	Pathopoiesia Pathopoiesia	Compound heterozygous
15 (27)	6Y	11Y	–	Chr3:49166502	15	c.1682C > A	P.R561Q	Clinical significance unknown	Heterozygous
16 (28)	55D	3 M	Nystagmus Small pupil	Ch3:49169117	5	C.499G > T	P.D167Y	Clinical significance unknown	Homozygous
17 (29)	18 M	18 M	High myopia	Chr3:49168239	8	C.970 T > C	P.C324R	Clinical significance unknown	Heterozygous
18 (17)	18 M	5Y	High myopia	Chr3:49168239	8	C.970 T > C	P.C324R	Clinical significance unknown	Heterozygous
19 (17)	36D	4 M	Retinal detachment	Ch3:49169080 Ch3:49163458–49,163,461	5 17	C.536C > T C.2283-2286delCTCT	p.S762RfsX28 p.N1325KfX1331	Pathopoiesia Pathopoiesia	Compound heterozygous
20 (30)	3Y	–	Small pupil Nystagmus	Ch3:49163404–49,163,475 Ch3:49160417	17 27	c.2269-2341del c.4249delA	p.S757cfs*11 P.D1415mfs*37	Pathopoiesia Pathopoiesia	Frame-shift
21 (23)	2Y	–	–	Ch3:49158666 Ch3:49162849	19 19	c.53890G > T c.2557C > T	p.C1797F p.R853X	Pathopoiesia Pathopoiesia	Compound heterozygous
22 (23)	8Y	–	–	Ch3:49150340 Ch3:49161830	19 17	C.4370G > A c.3335G > A	p.R1457Q P.E1109K	Clinical significance unknown Clinical significance unknown	Compound heterozygous
23 (23)	3 M	–	–	Ch3:49163567	17	c.5182C > T	pGln1728∗	Pathopoiesia	Compound heterozygous
24 (24)	1Y	–	–	Ch3:49164326	17	c.2890C > T	pArg964∗	Pathopoiesia	Compound heterozygous
25 (20)	1 M	3 M	–	Chr:49168561	7	c.737G > A	p.R246Q	Clinical significance unknown	Homozygous
26 (20)	7D	4 M	–	Chr:49168561	7	c.737G > A	p.R246Q	Clinical significance unknown	Homozygous

## Results

**Figure 1:**
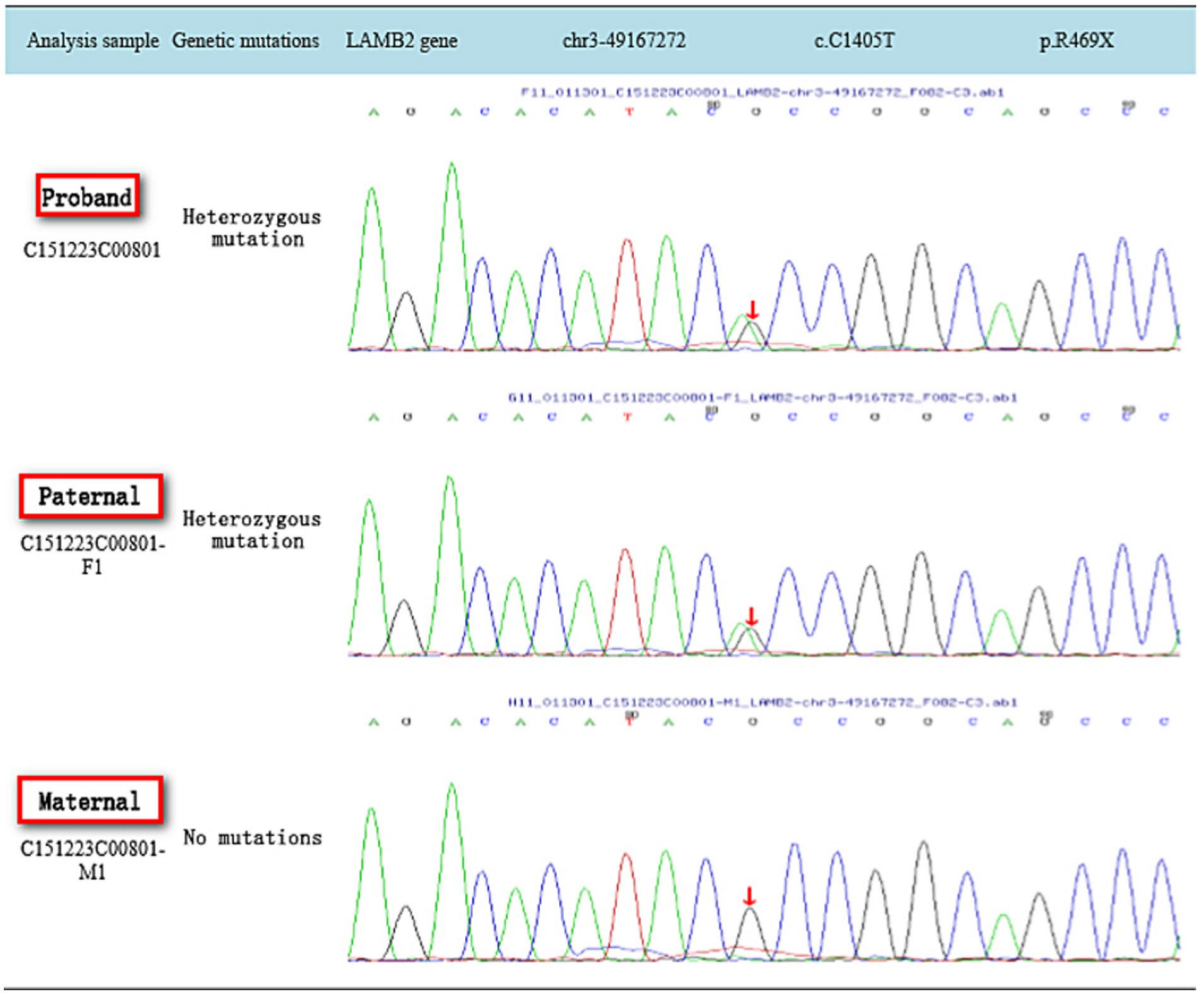
Gene sequencing map of family c.C1405T.

**Figure 2:**
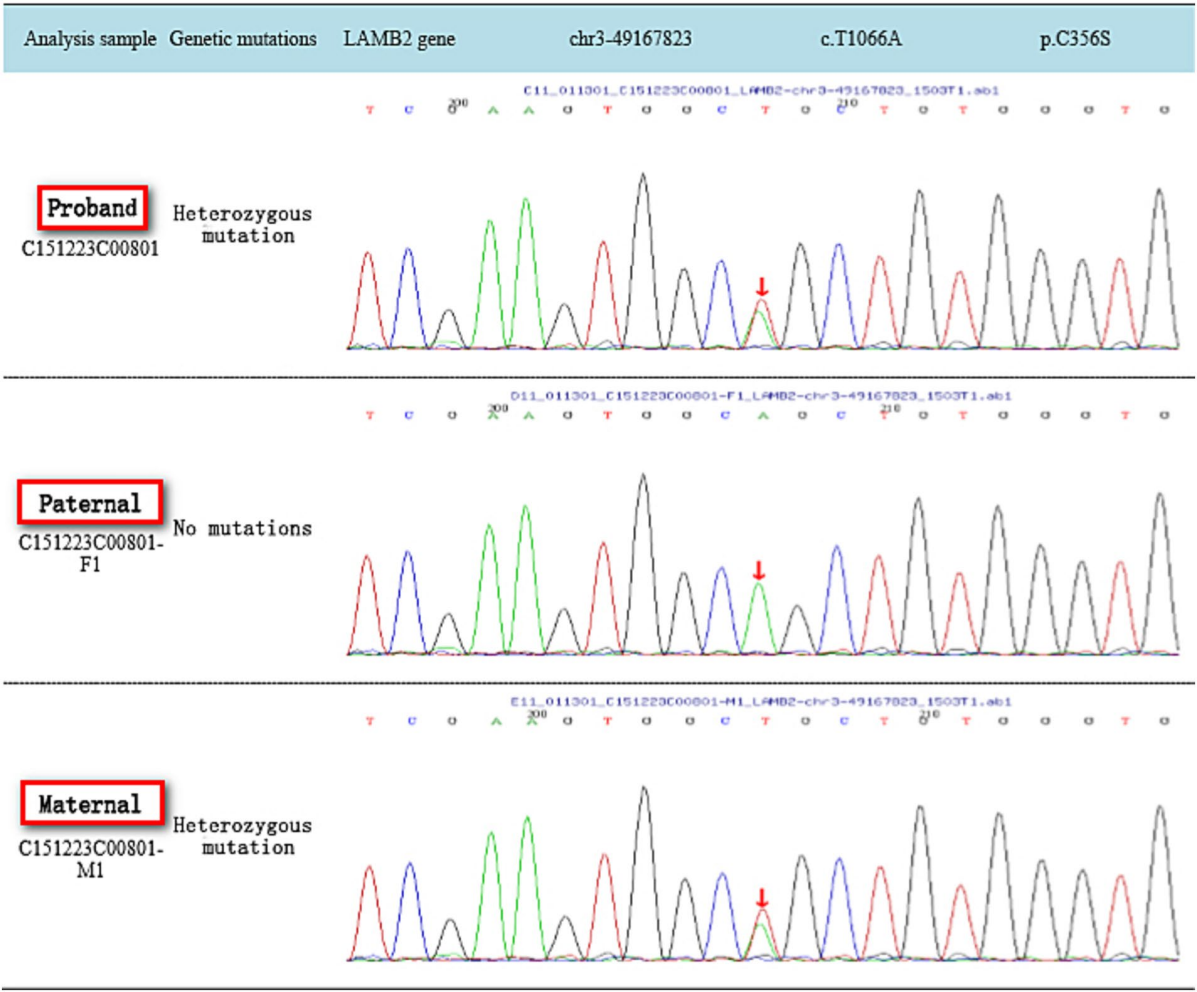
Gene sequencing map of family c.T1066A.

1. The case report describes the phenotype and genotype of a female patient, aged 9 months, who presented with “edema and massive proteinuria,” and serum albumin was 14 g/L, serum creatinine was 12 μmol/L, serum choleterol was 5.79 mmol/L, urine protein/creatine ratio was 6.5, diagnosed with “nephrotic syndrome.” The patient exhibited small pupils and horizontal nystagmus, with no notable history at birth. She is the second child of her mother and there is no family history of similar conditions. Despite 4 weeks of high-dose steroid (2 mg/kg*d) induction therapy, her proteinuria remained unresolved, indicating steroid resistance. Subsequently kidney pathology and genetic testing were conducted. The kidney pathology revealed minimal change glomerular disease. Cyclosporine was added to her steroid regimen. One month later, genetic testing revealed two heterozygous mutations in the *LAMB2* gene, one inherited from the father and the other from the mother, leading to a diagnosis of Pierson syndrome. After 1 month of treatment, the serum albumin was 13 g/L, serum creatinine was 34 μmol/L, serum choleterol was 5.89 mmol/L, urine protein/creatine ratio was 6.2, consider treatment ineffective, we stopped using cyclosporine and reduced the dosage and stopped using steroids within 1 month. Despite ongoing treatment, the proteinuria persisted, and at the age of 5 years and 11 months, the patient developed chronic kidney disease stage 5, necessitating dialysis.
2. The full exome sequencing results of the reported case: Compound heterozygous mutations in the *LAMB2* gene c.1405C > T (p.R469X) and c.1066 T > A (p.C356S) were identified, each inherited from the father and mother, respectively. Both mutations were previously unreported and considered novel variants. According to the ACMG guidelines for the classification of genetic variants, the *LAMB2* gene c.C1405T (p.R469X) variant was classified as likely pathogenic (PVS1 + PM2), and the *LAMB2* gene c.T1066A (p.C356S) variant was classified as of uncertain significance (PM2 + PP3; as shown in Figures 1, 2).
3. Literature review: 21 published articles reported 26 cases of nephrotic syndrome resulting from *LAMB2* gene mutations, with 18 of these cases undergoing kidney biopsy. The biopsy results indicated focal segmental glomerulosclerosis in 10 cases, minimal change disease in 4 cases, diffuse mesangial sclerosis in 2 cases, IgM nephropathy in 1 case, and mesangial proliferation in 1 case. Electron microscopy revealed basement membrane splitting in 10 of these patients. Genetically, 15 cases exhibited compound heterozygous mutations, five had homozygous mutations, three had heterozygous mutations, two had frameshift mutations, and one had a truncating mutation.

26 cases of pediatric patients were reported, with 14 experiencing ocular symptoms. Among these, 16 cases developed end-stage kidney disease (ESRD), all presenting as either steroid-resistant nephrotic syndrome or congenital nephrotic syndrome, the median age for ESRD is 4 (2, 18) months, with minimum age of 28 days and maximum age of 16 years. Among the five cases with homozygous mutations, all progressed to ESRD during infancy. Additionally, three cases with compound heterozygous *LAMB2* mutations were described. All these exhibited steroid-resistant nephrotic syndrome and failed to respond to steroid therapy, advancing to ESRD within 1–2 years after diagnosis. Out of the 15 cases with compound heterozygous mutations, seven eventually developed ESRD. One case with a truncating mutation also advanced to ESRD in infancy, while two cases with frameshift mutations did not progress to ESRD within the reported timeframe.

15 out of the 26 patients had concurrent extrarenal symptoms. The most frequent of these were miosis and nystagmus, affecting four cases, respectively. Other manifestations included visual impairment in three cases, epidermolysis bullosa in one case, retinal detachment in one case, and other ocular abnormalities in one case.

Among the 26 patients, 10 displayed basement membrane lesions that were characterized by layering and tearing, without a prominent saw-tooth structure or uniform thickness. Within this group, four cases presented homozygous mutations with genetic alterations identified as c.1890G > T, c.737G > A, c.970 T > C, and c.499G > T. The remaining six cases had compound heterozygous mutations, with gene mutations listed as c.235-237delGTC, c.737G > A, two instances of c.53890G > T, c.4370G > A, and c.5182C > T. All four patients with homozygous mutations progressed to ESRD during infancy. The other six cases, presenting in the preschool and early childhood periods, did not have their specific timing of progression to ESRD detailed in the literature.

## Discussion

The glomerular basement membrane (GBM), along with the glomerular capillary endothelial cells, the endothelial cell surface membrane, the visceral layer of Bowman’s capsule podocytes, and the podocyte foot process slit diaphragm, constitutes the glomerular filtration barrier. Extensive studies have demonstrated that alterations in the components, structure, and thickness of the GBM are intimately linked to the development and progression of various glomerular diseases, including metabolic kidney diseases, hereditary kidney diseases, and immune-mediated glomerulonephritis (10). The GBM, which is a specialized extracellular matrix situated between the endothelial cells and the podocytes, is composed of type IV collagen, laminins, nidogens, and heparan sulfate proteoglycans. Mutations in the *LAMB2* gene disrupt the synthesis or function of the laminin α5β2γ1 (LM-521) isoform, resulting in Pierson syndrome, which is characterized by congenital nephrotic syndrome alongside eye and neurological defects. However, milder variants of these mutations may manifest solely as congenital nephrotic syndrome without any extrarenal symptoms (11).

Laminin, a heterotrimeric glycoprotein found in all basement membranes, is composed of various *α*, *β*, and *γ* chains, with LM-521 being the primary form in the GBM (12). Pierson syndrome, an autosomal recessive disorder, arises from mutations in the *LAMB2* gene located on chromosome 3, which encodes the laminin β2 subunit consisting of 32 exons and 1798 amino acids. Laminins are essential components of the basement membrane (13), and play a vital role in cell adhesion, proliferation, differentiation, and migration. The laminin β2 subunit, predominantly located in the GBM, ocular structures, and neuromuscular synapses, is critical for structural integrity. Mutations in *LAMB2* result in the absence of the laminin β2 subunit, a key element of the GBM, leading to the manifestation of Pierson syndrome.

Typically, Pierson syndrome manifests as congenital nephrotic syndrome, accompanied by non-reactive pupils and neuromuscular deficits, with patients frequently progressing to end-stage kidney disease in early infancy. Ocular involvement is a significant symptom of the condition, with miotic pupils being the most prevalent ocular symptom. Additional ocular abnormalities reported include iris abnormalities, cataracts, lens shape abnormalities, retinal abnormalities, and high myopia. Recently, mutations in the *LAMB2* gene have been identified in patients with kidney disease who lack noticeable extrarenal manifestations. Some of these cases have retained normal kidney function into adolescence, contrasting sharply with the typical progression of Pierson syndrome. This study also noted that 15 out of 26 children with Pierson syndrome exhibited extrarenal symptoms. The authors suggest that ocular abnormalities may develop as the disease progresses, necessitating thorough ophthalmological assessments during follow-up care. The findings indicate that these patients might represent an atypical variant of Pierson syndrome, and it has been proposed by international researchers that Pierson syndrome can occur with nephrotic syndrome alone, without neurodevelopmental or ocular abnormalities.

According to international researchers (3), the kidney pathological features of Pierson syndrome are as follows: light microscopy reveals an increased mesangial matrix without mesangial proliferation or complete sclerosis; podocytes may exhibit an immature cuboidal fetal appearance; glomerular cystic changes are observable; and there is proportional interstitial atrophy and fibrosis of the tubules. Routine immunofluorescence staining does not reveal immune complexes, and additional research has confirmed the absence of the β2 chain of the laminin protein in classic Pierson syndrome, whereas in cases of Pierson syndrome presenting solely with nephrotic syndrome, laminin β2 chain expression is diminished. Electron microscopy demonstrates an increase in mesangial matrix without deposition; podocytes display extensive foot process effacement, and the glomerular basement membrane exhibits irregular thickening and thinning. Among the 18 patients in this study, mesangial cell and matrix proliferation, focal segmental glomerulosclerosis (pericapsular type), and minimal change glomerular disease were also noted. These varied pathological changes may correlate with the age at onset, duration of the disease, and severity of clinical symptoms at the time of kidney biopsy.

The current purpose of treating GBM-related diseases is to reduce intraglomerular pressure and treat the underlying causes as much as possible (14). As our knowledge of GBM maintenance and replacement evolves, novel therapeutic approaches, such as those replacing GBM components or stimulating GBM repair, may emerge as new treatments for patients with GBM-associated disorders (14). Currently, the management of Pierson syndrome is primarily supportive, with no specific effective treatments available. Supportive care includes managing water and electrolyte balance, administering albumin infusions, and providing dialysis treatment when necessary (5). When these patients experience progressive kidney deterioration, an early evaluation for kidney transplantation is recommended (5).

For children diagnosed with congenital nephrotic syndrome or steroid-resistant nephrotic syndrome, it is crucial to thoroughly assess the involvement of additional organs, including skeletal, nervous system, eyes, ears, and the urogenital system for malformations. The International Pediatric Nephrology Association recently issued new global guidelines for the diagnosis and management of pediatric steroid-resistant nephrotic syndrome. These guidelines advocate for a kidney biopsy in all cases of steroid-resistant nephrotic syndrome, except when the condition is clearly secondary to infections or malignancies, or when the patient presents with familial, syndromic, or genetic forms of the disease. Additionally, genetic testing is recommended for all cases of primary pediatric steroid-resistant nephrotic syndrome to prevent delays in diagnosis and unnecessary treatments.

## Data Availability

The datasets presented in this study can be found in online repositories. The names of the repository/repositories and accession number(s) can be found in the article/supplementary material.

## Footnotes

^1^broadinstitute.org/
